# Automated Technique for Brain Tumor Detection From Magnetic Resonance Imaging Based on Local Features, Ensemble Classification, and YOLOv3

**DOI:** 10.1155/bmri/5531209

**Published:** 2025-11-29

**Authors:** Danish Arif, Zahid Mehmood, Amin Ullah, Ahmad Fawad, Simon Winberg

**Affiliations:** ^1^ Department of Electrical Engineering, University of Cape Town, Rondebosch, South Africa, uct.ac.za; ^2^ Department of Computer Engineering, University of Engineering and Technology, Taxila, Pakistan, uet.edu.pk; ^3^ Department of Computer Science, Bahria University, Lahore, Pakistan, bahria.edu.pk; ^4^ Faculty of Information Technology, University of Central Punjab, Lahore, Pakistan, ucp.edu.pk

**Keywords:** compute unified device architecture (CUDA), computer-aided diagnosis (CAD), digital imaging and communication in medicine (DICOM), gray-level co-occurrence matrix (GLCM), magnetic resonance imaging (MRI), parallel computing

## Abstract

In this article, the researcher explores an automated approach for detecting a brain tumor using MRI scans of the brain. In underdeveloped countries, many people are dying due to the slow detection process and other negligence of radiologists. People suffer from these diseases due to the slow process of recognition. Since the number of patients is greater than that of radiologists, there is the possibility of human error, which can cause serious damage. The detection of tumors from magnetic resonance imaging (MRI) data is an important manual task, specifically in terms of the time that the radiologist performs. In this study, the researchers sought to study state‐of‐the‐art techniques to detect normal brain and brain tumors from MRI using machine learning techniques. The main objective of this study is to develop a novel automated technique for brain tumor detection. Through the worldwide consideration of practical literature, it is clear that traditional approaches are insufficient to resolve all uncertainties and problems. Therefore, a novel approach to examining MRI must be adapted. This study proposes two different novel techniques: one that uses ensemble classification and the other that makes use of the deep learning model of YOLOv3. In ensemble classification, two classification algorithms are used which are support vector machine (SVM) and K‐nearest neighbors (KNNs). The YOLOv3 model is used to detect and outline tumor locations in the images. This study used an open‐source dataset and data collected from hospitals in Lahore, Pakistan. The ensemble classifier achieved an overall accuracy of 80.50%, while the YOLOv3 model achieved higher performance with 97.80% accuracy, 97.40% precision, 98.18% recall, and a mean intersection over union (IoU) score of 0.65. These results confirm that YOLOv3 is a useful technique for identifying brain tumors.

## 1. Introduction

In Pakistan, more than 100,000 people die due to cancer [1]. Oral, breast, and brain cancers are the most common cancers in Pakistan. Brain cancer is responsible for only 2% of all cancer deaths in Pakistan. Brain and other nervous system cancers are the 10th leading cause of death for men, women, and children. The statistical information on malignant glioblastomas is more critical and unadorned since its survival rate is only 2 years. The only solution is to surgically remove the tumor when you see the painful and overwhelming mishaps and the results of the tumors. In Pakistan, many patients die due to the slow process of detection and other negligence of radiologists. A lot of people are suffering from these diseases due to the slow detection process. As the number of patients is more than the number of radiologists, there is a chance of human error which can cause serious damage. Radiologists identify tumors from magnetic resonance imaging (MRI) data, and it is very time‐consuming [2, 3].

Brain tumor identification is an important process for extracting complex MRI information from brain images. The automated detection method has greatly improved knowledge of normal tests and diseases in medical research and plays an important role in diagnosis and treatment planning as the number of patients increases. Doctors and radiologists diagnose tumors using MRI, which is a noninvasive medical test. MRI provides accurate images of structures, loose tissues, bones, and almost all structures and internal organs of the body through a strong magnetic field, radio waves, and a computer. MRI shows a clear vision of the brainstem and the posterior brain, which is problematic for the understanding of CT scans [4, 5].

The diagnosis and analysis of the tumor rely on manual results and medical tests. This is hard to manage when there are fewer doctors and plenty of patients waiting to get results and diagnoses. In addition, an automatic system or tool for MRI analysis is needed to decrease the risk of misdiagnosis. Doctors spend most of their time diagnosing this instead of giving their patients enough time [6]. This study develops an automated diagnosis system to help doctors in error‐free diagnosis of tumors in less time. This automation is achieved by detecting the area of the tumor using advanced algorithms. When the same task is performed by a doctor, it requires effort and time. The proposed technique reduces the burden on doctors. This research includes the detection and classification of brain tumors using the established classifier. MRI datasets consisting of normal brain MRI and tumors are used, and a model identifies and calculates the area of the tumor part when the patient has a brain tumor.

The major contributions of the proposed technique are as follows:

1. The main motive of this study is to guide automated diagnosis approaches and systems that help doctors to diagnose brain tumors, reduce the likelihood of erroneous diagnoses, and save time using machine learning (ML) algorithms.
2. We have also analyzed the main deficiencies of the existing methods for brain tumor detection, that is, less accurate results, speed, area limits, and inability to distinguish between the normal brain and a brain having a tumor in MRI data.
3. This study proposed two novel techniques based on YOLOv3 and ensemble classification for effective brain tumor detection from MRI data. For optimal feature extraction, the proposed technique uses statistical features and gray‐level co‐occurrence matrix (GLCM) features.
4. Experimental analysis of both techniques concludes that the proposed technique based on the YOLOv3 outperforms in terms of standard performance evaluation metrics for brain tumor detection from MRI data.

The remaining sections of this article are organized as follows: Section 2 presents the details of the related works of brain tumor detection from MRI data. Section 3 presents the details of the methodology of the proposed technique. Section 4 presents the results and discussions of the proposed technique, and Section 5 concludes the proposed technique and presents the future work.

## 2. Related Works

Brain tumor detection and classification pose significant challenges to both patients and medical professionals due to their diverse types, complex morphologies, and potential for rapid progression. Timely and accurate detection, as well as classification of these tumors, is critical for effective treatment planning and patient management. However, traditional methods for brain tumor diagnosis, such as manual interpretation of medical imaging, are often time‐consuming, subjective, and prone to human error.

In [7], the authors propose a deep learning (DL)–based method for both classifying and segmenting brain tumors using a multiscale convolutional neural network (CNN). The input MRI images are preprocessed to enhance their quality and standardize their appearance, making them suitable for analysis. A multiscale CNN is employed to automatically extract relevant features from the preprocessed MRI images. This CNN architecture likely incorporates multiple layers with different receptive fields to capture features at different scales. The extracted features are fed into a classification model, such as a SoftMax classifier, to classify the brain images into different tumor types (e.g., glioma, meningioma, and metastatic tumor). The approach also segments the tumors within the images. This segmentation process involves delineating the boundaries of the tumors to accurately identify their location and extent within the brain. The performance of the method is evaluated using various metrics such as accuracy, sensitivity, specificity, and Dice coefficient. These metrics assess how well the model classifies tumors and accurately delineates their boundaries compared to ground truth annotations. Lavanyadevi et al. [8] introduce a method for brain tumor classification and segmentation in MRI images using probabilistic neural networks (PNNs). They address the challenge of accurately classifying and segmenting brain tumors from MRI images, which is crucial for diagnosis and treatment planning. This method utilizes PNN to classify data based on probability distributions. PNNs are particularly suitable for classification tasks involving complex and nonlinear relationships between input features. The MRI images are preprocessed to enhance their quality and normalize their intensities, ensuring consistent input for the neural network. The texture, shape, and intensity features are extracted from the preprocessed MRI images. The extracted features are fed into the PNN for classification into different tumor categories, such as glioma, meningioma, or metastatic tumor. The PNN assigns a probability distribution to each class, allowing for uncertainty estimation in the classification. In addition to classification, the method is aimed at segmenting the tumors within the MRI images. This involves delineating the boundaries of the tumors to precisely localize and measure their extent within the brain. By incorporating probability distributions and considering uncertainty, the method offers a robust approach to assist in clinical decision‐making for brain tumor diagnosis and treatment planning.

Solanki et al. [9] explore the application of ML and DL techniques in categorizing brain tumors. The research is aimed at enhancing the accuracy and efficiency of tumor classification for improved diagnosis and treatment planning. The study employs various ML algorithms such as support vector machines (SVMs), decision trees, and random forests, along with DL models like CNNs and recurrent neural networks (RNNs). Data preprocessing techniques such as feature extraction and dimensionality reduction are also utilized to enhance model performance. The results demonstrate promising accuracy rates in classifying brain tumors, showcasing the potential of ML and DL approaches in medical image analysis and diagnosis. In [10], the authors provide a comprehensive review of various intelligence techniques applied in the detection and classification of brain tumors. The study covers a range of methodologies including ML, DL, and other artificial intelligence (AI) approaches. It discusses the advancements in medical imaging technologies such as MRI and CT scans, which serve as primary sources for tumor detection. The overview discusses the challenges associated with accurate tumor classification and emphasizes the importance of feature extraction, selection, and fusion techniques in improving classification accuracy. Additionally, it highlights the significance of large‐scale datasets and the role of different algorithms such as SVMs, artificial neural networks (ANNs), CNNs, and hybrid models in enhancing the performance of tumor detection and classification systems. The overview serves as a valuable resource for researchers and practitioners in the field, providing insights into the current state‐of‐the‐art techniques and future directions for advancing brain tumor diagnosis and treatment.

Naser and Deen [11] introduce a DL‐based approach for the segmentation and grading of lower grade gliomas (LGGs) in MRI images. They developed an automated method to segment LGG tumors from MRI images and assign them a grade based on their characteristics. This method employs a DL architecture based on a CNN for image analysis to capture complex spatial features. The MRI images are preprocessed to enhance their quality and standardize their appearance. The DL model is trained to segment LGG tumors within MRI images to accurately delineate the boundaries of the tumors to identify their location and extent within the brain. Once the tumors are segmented, the model assigns them a grade based on their characteristics. In the case of LGGs, grading involves assessing factors such as tumor size, shape irregularity, and enhancement patterns on MRI images to determine the malignancy level. The performance of this method is evaluated using various metrics such as the Dice coefficient, sensitivity, specificity, and accuracy. These metrics assess how well the model segments tumors and grades them compared to ground truth annotations provided by medical experts. The approach in [12] focuses on the application of DL techniques for the detection and classification of brain tumors. This approach utilizes a DL classifier, which is a type of ANN capable of learning complex patterns from data. The research involves the preprocessing of medical imaging data, such as MRI scans, to extract relevant features that can aid in tumor detection and classification. These features are then used as input to the DL classifier, which is trained on a dataset containing labeled brain tumor images. It evaluates the performance of the DL classifier in terms of accuracy, sensitivity, specificity, and other relevant metrics. It compares the results with existing techniques to demonstrate the effectiveness of the proposed approach. It contributes to the development of more accurate and efficient methods for diagnosing brain tumors using DL techniques, potentially improving patient outcomes through earlier detection and classification of tumors. The technique in [13] investigates the efficacy of DL methods in diagnosing brain tumors through the analysis of MRIs. The study systematically reviews various DL techniques employed in the diagnosis of brain tumors and evaluates their performance in comparison to traditional methods. It highlights the potential of DL techniques in revolutionizing the diagnosis and management of brain tumors by providing accurate, efficient, and reliable solutions for medical professionals.

In [14], the authors presented a hybrid classification approach for brain tumor detection using MRI images. The authors performed classification by using an ensemble deep neural support vector machine (EDN‐SVM) model. This hybrid model combines the strengths of neural networks and SVMs. It extracts features automatically and enhances classification. The authors have compared the EDN‐SVM model with other solo conventional models such as CNN, RFC, ANN, and R‐CNN, but the proposed method performed best among all, with an accuracy of 97.93% for classifying normal and abnormal brain tissues. The research in [15] created a new model SEL‐DenseNet201, which combines the strengths of some existing computer models. It was trained on brain MRI images, and the authors used different methods to improve image quality and balance the data. The new system achieved nearly perfect accuracy of 99.65% for finding tumors precisely. In [16], the authors used the BRATS 2020 dataset for training a CNN in segmenting and classifying brain tumors. As the dataset contains four types of tumor data, this model shows high accuracy in segmentation and classification of MRI images. In [17], the authors used InceptionV3 and YOLOv2 as a combined model for locating tumors in images. This method is tested on three versions of BRATS data, showing the best results on the latest data.

The obstacles associated with the aforementioned methods for detecting brain tumors include separate stages for detection and segmentation which increases computational complexity and inference time. The proposed technique of YOLOv3 for brain tumor detection in MRI scans is significant because it allows for quick, automated, and precise tumor localization, which facilitates early diagnosis and prompt medical treatment. By using real‐time object detection to quickly and accurately identify tumor locations, YOLOv3 improves diagnostic efficiency in contrast to traditional manual analysis, which is laborious and prone to human error. Its computationally fast single‐shot detection architecture makes it appropriate for incorporation into clinical workflows, particularly in environments with restricted resources. By automating initial screens, YOLOv3 lessens the workload of radiologists, speeds up decision‐making, and improves patient outcomes by identifying potentially fatal abnormalities early. It is a useful tool for early tumor detection in extensive medical imaging applications due to its speed and accuracy balance.

### 2.1. Preliminaries

#### 2.1.1. Brain Tumors

Brain tumors are masses of cells that have developed and multiplied by uncontrollable brain tumors. It is an uncontrolled growth of the solid mass of unwanted cells that are commonly desired in different parts of the brain, such as glia, neurons, and lymphoid tissue. The blood contains blood vessels, pituitary, pineal, cranium, or tumors, which are disseminated mainly in other organs. Brain tumors are classified according to the type of tissue involved in the brain, the location of tumors in the brain, benign tumors, or other factors. Brain tumors are solid components that enter the surrounding tissues or alter the surrounding structure. There are different types of brain tumors: (i) glioma, (ii) medulloblastomas, (iii) lymphoma, (iv) meningioma, (v) craniopharyngioma, and (vi) adenoma of the pituitary gland [18, 19].

#### 2.1.2. ML

**Figure 1:**
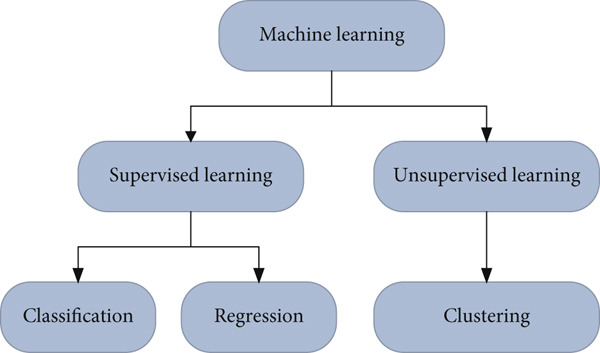
Categories of machine learning approaches.

ML is a branch of learning that creates a statistical model by combining computer ideologies and statistics and then tells the computer what logically reaches the different creatures [20, 21]. Subsequently, implications and estimates are made to determine the contours and models of this statistical information and to predict future results based on previous data [22]. It consists of different categories as shown in Figure 1 to extract data from the provided information without relying on previously defined calculations and prototypes. We apply ML when we have a lot of information and different sets but no defined formulas and methods [23].

##### 2.1.2.1. Supervised Learning

It is an algorithm that uses the recognized records for prophecy. The known datasets consist of input information and related answers. Supervised learning predicts the outcome of an unknown dataset. The authenticity of the design is often confirmed by the test dataset. An accurate forecast can be made if we use a large number of recognized and trained records [24, 25]. This procedure consists of two major categories: classification and regression. The classification predicts discrete results that interrupt the information in specific groups while regression is used for consistent or continuous value results. The most widely used classification and regression methods for brain tumor classification are mentioned in Table 1.

**Table 1:** Mostly used classification methods for brain tumor classification.

**Classification**	**Regression**
Neural networks	Nonlinear regression
Support vector machine	Linear regression
Decision trees	Decision trees
Discriminant analysis	Generalized linear models

In this study, we have used ensemble classification and YOLOv3 for the proposed technique by taking training data from the CSV file, and after that, based on the combined results, the test image is labeled. The details of the two different classifiers (i.e., SVM and KNN) used for ensemble classification are as follows.

###### 2.1.2.1.1. SVM Classifier

The SVM is a supervised ML algorithm that can be used for classification and regression tasks but mainly for classification problems. In this algorithm, we plot each data element as a point in dimension *n*. In the space (where *n* is the number of entities present) with the value of each entity as a given coordinate value, we classify and look for a hyperplane that distinguishes two classes very well [26].

###### 2.1.2.1.2. K‐Nearest Neighbor Classifier

The K‐nearest neighbor is the data classification algorithm that attempts to determine by observing the surrounding data points, the location of the data collection group. The algorithm verifies the point in the raster and tries to determine if a point belongs to the Group “A” or if the state of the near point is verified. The range is determined arbitrarily, but it is about sampling the data. If most of the eye belongs to Group “A,” the probable point of the data point is “A,” not “B,” and vice versa. The nearest neighbor *k* is a good example of a good learning algorithm because it does not produce a model for previous records. The only calculations he made were when asked to look at the neighbors from the data points [27].

###### 2.1.2.1.3. Ensemble Classifier

In ensemble classification, the results of ML can be improved by combining different classification models and creating a new model, the overall classification model. This method allows for accurate prediction and better results, rather than applying a single model. The ensemble model is a meta‐algorithm that combines many algorithms and ML methods into a single predictive method to minimize variance and distortion and improve prediction [28].

##### 2.1.2.2. Unsupervised Learning

It is an algorithm in which the implication occurs using recognized and known datasets, including input information, but without characterized results. In this procedure, the cluster analysis performs a preliminary analysis to determine the hidden forms and categories in the information provided by [29, 30]. In this category, different grouping techniques are used for brain tumor classification as mentioned in Table 2.

**Table 2:** Different clustering methods for brain tumor classification.

**Clustering**
*k*‐means clustering
Hidden Markov models (HMMs)
Self‐organizing masks
Gaussian mixture models (GMMs)
Hierarchical clustering

##### 2.1.2.3. DL

The ML has a subfield called DL. It is hierarchical and has deep structured learning. The human‐like ANN techniques are used in DL [31]. DL utilizes computational models composed of multiple layers to process data, enabling the learning of intricate representations through various levels of abstraction. This approach has significantly enhanced AI tasks such as image and speech recognition, object detection, and even applications in genetics. With large datasets, the interconnections between nodes within these layers can become exceedingly complex, posing challenges across all layers. However, DL addresses this issue through the backpropagation algorithm, which enables machines to determine how internal parameters should be adjusted across layers to compute each layer’s representation effectively [32]. DL employs diverse architectures such as CNNs, deep belief networks, deep recurrent networks, and deep neural networks. These architectures find application across a wide range of fields including bioinformatics, machine translation, computer vision, speech recognition, medical imaging, and material analysis. DL has delivered impressive outcomes in these domains, sometimes even outperforming human capabilities [33].

#### 2.1.3. Parallel Computing

The parallel computing involves the simultaneous use of multiple computing resources to solve computational problems. The problems are divided into several parts that can be solved simultaneously. Each section is divided into a series of instructions. The instructions on each side run simultaneously on different processors. A complete control/coordination mechanism is used.

#### 2.1.4. Compute Unified Device Architecture (CUDA)

The CUDA is a parallel computing platform and a programming model developed by Nvidia for general computing. CUDA allows developers to accelerate computationally intensive applications using the power of the graphics processing unit (GPU) to calculate parts that can be paralleled [34].

## 3. Methodology

### 3.1. MRI of the Human Brain

**Figure 3:**
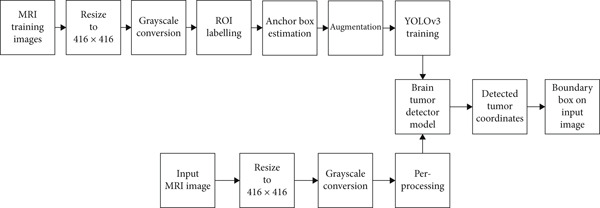
Block diagram of the proposed technique based on the YOLOv3 for brain tumor detection.

The magnetic resonance scans use magnets and powerful radio waves to create detailed images of the human body. In human tissues, hydrogen molecules, water molecules, polarization, and protons produce space spatial signals [35–37]. The magnetic resonances mainly use three electromagnetic fields: (i) a very strong static magnetic field for the polarization of the hydrogen nuclei, called the static field; (ii) a lower temporal variation field for the spatial coding, called the gradient field; and (iii) a high‐frequency field that allows the hydrogen nucleus to generate a signal that can be measured through an RF antenna. The behavior of the proton variables in different tissues causes variations in the appearance of the tissues. The MRI of differential brain position is shown in Figure 2a with the weight of T1 and T2 while an MRI of a brain tumor in the human brain is shown in Figure 2b. The block diagram of the proposed technique based on the YOLOv3 is shown in Figure 3.

Figure 2(a) MRI of the human brain. (b) MRI of a brain having a tumor in the human brain.

**Figure:**
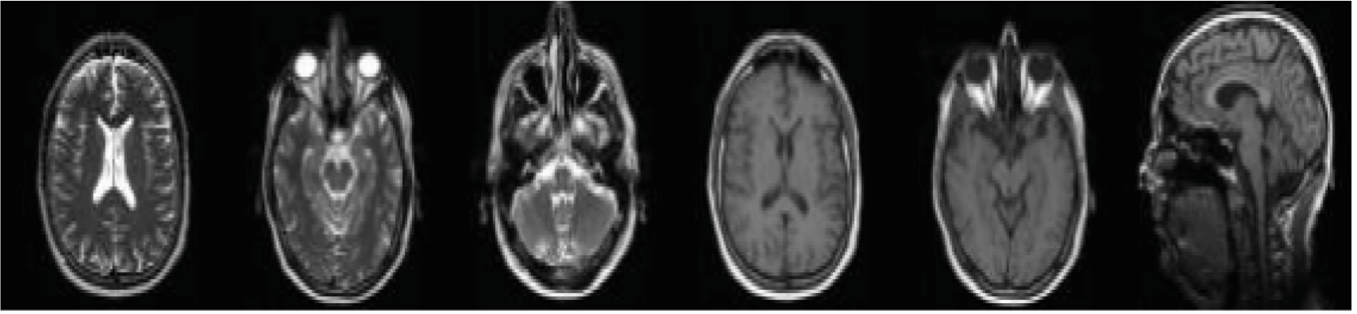
(a)

**Figure:**
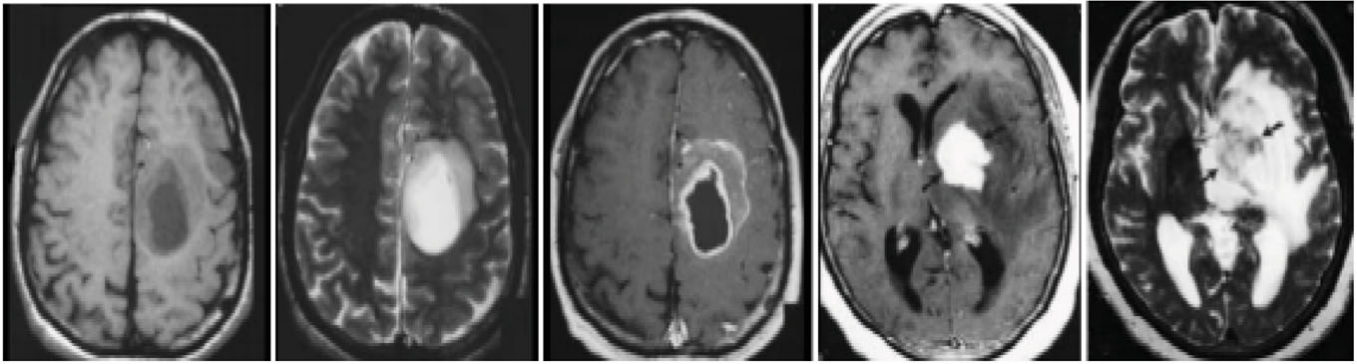
(b)

According to the details shown in Figure 3, there are two steps in developing and using the AI model: one is training the model, and another is using the model for prediction. In the process of training, MRI images for the dataset are gathered from both the INMOL and General Hospital in Lahore, Pakistan, under the careful supervision of experienced radiologists. The second step is to resize the MRI images as per the input layer of the proposed DL model of YOLOv3. The YOLOv3 model consists of multiple options for input sizes; the proposed technique based on YOLOv3 uses an image size of 416 × 416 pixels. As the color in MRI images is not a required feature by the proposed technique, the images are converted into grayscale. YOLOv3 requires labeled data that tells the region of interest for training purposes. All the images are carefully labeled into two classes: with and without tumors. The proposed technique based on the YOLOv3 introduced a new parameter known as an anchor box; these anchor boxes are calculated to estimate the size of potential detected tumors in positive images. The total numbers of images which are present in the dataset are increased with the help of an augmentation technique which uses flipping, variation in brightness, and angles. With the help of the augmentation, the proposed technique became more robust, accommodating MRI images from different types of hardware. The details of the training parameters of the proposed technique are mentioned in the training section. In the detection (testing) phase, the input image is resized to 416 × 416 pixels and then converted into a grayscale image. The same preprocessing is done as for the ensemble classification model. The resultant image is fed to pretrained YOLOv3 technique. It returns the coordinates for the detected tumor(s). The boundary box is created around the tumor(s) using these coordinates.

**Figure 4:**
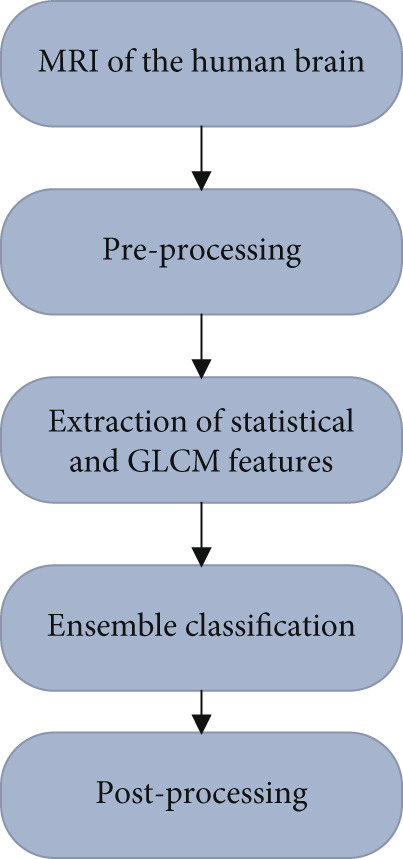
Block diagram of the proposed technique based on the ensemble classification for brain tumor detection.

**Figure 5:**
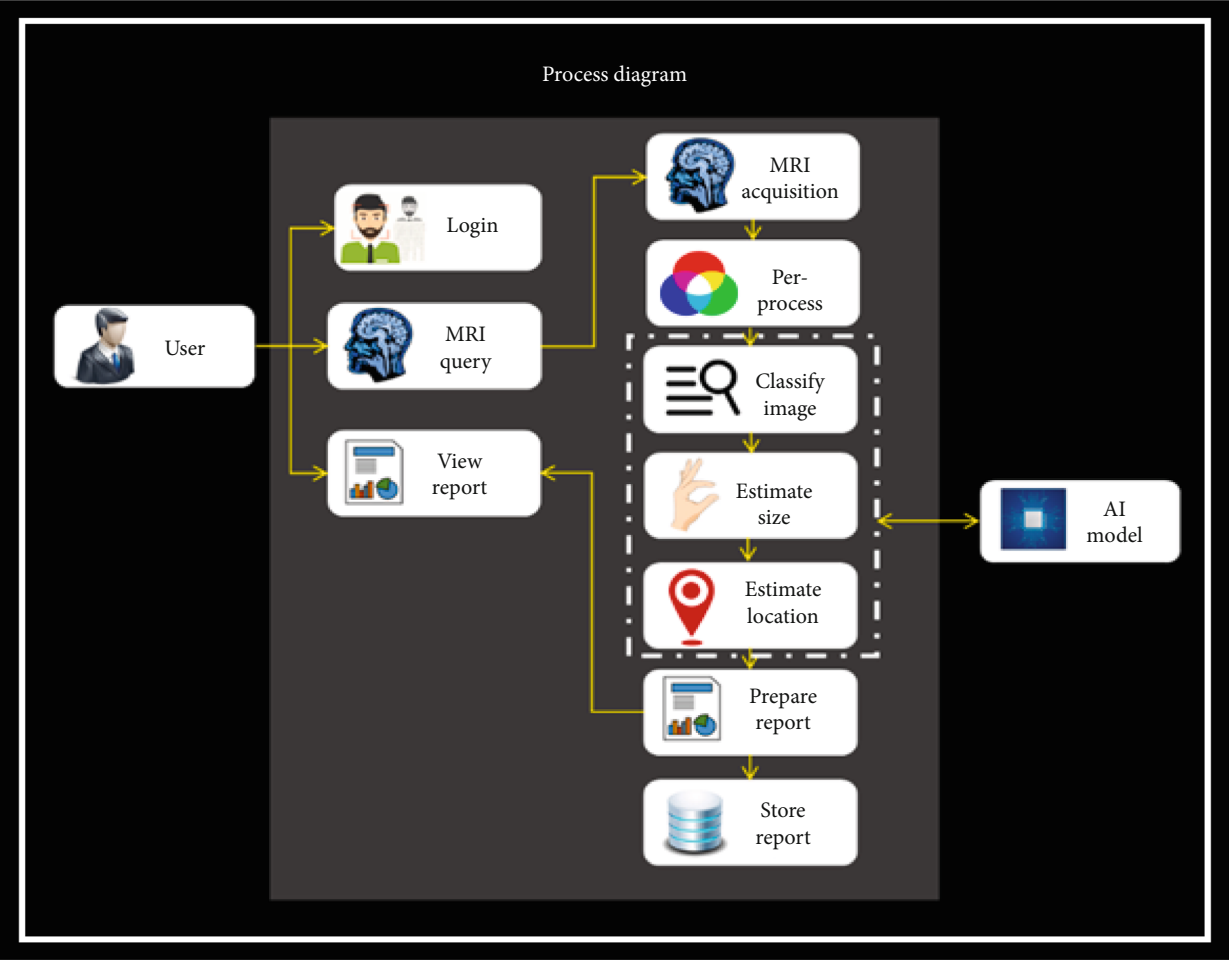
Process diagram of the proposed technique.

The proposed technique also introduced a novel computer‐aided diagnosis (CAD) system based on ensemble classification which is an automated system for brain tumor detection and classification through MRI. The CAD system of the proposed technique takes an image, classifies it, and estimates the area and location if there is a tumor in an image. An automated brain tumor detection procedure of the proposed technique follows some steps which are shown in Figure 4, whereas a process diagram of the proposed technique is shown in Figure 5.

The MRI brain image will be an input to the CAD which will be of two types: (i) MRI scan of a brain tumor image and (ii) MRI scan of a healthy brain. The MRI of the brain image that we get is in the DICOM form which contains an image and the details of the patient. The DICOM images more commonly use the (.DCM) extension. While the .DCM file is in 16‐bit format, it can vary from machine to machine. After getting the .DCM file, the image is converted according to the format of the gathered dataset.

### 3.2. Collection of the Data

AI has been widely used in daily life problems in various domains. A similar trend has been observed in medical imaging and other signal analysis tasks. Automatic brain tumor detection has been performed using a U‐Net [38]. CNN‐based brain tumor segmentation has been performed by [39] on the BRATS dataset. It contains enhanced MRI images and is organized into four categories: glioma tumor, meningioma tumor, pituitary tumor, and no tumor. Each category contains almost 500 images. The images are in JPEG format with resolutions around 512 × 512 pixels and include axial, sagittal, and coronal views. Similarly, DL‐based segmentation models have been introduced. Now the concern is with the images from the older MRI machines having artifact complications. The proposed technique is applied to the data acquired from INMOL hospital which has such issues. The dataset comprising DICOM images of brain MRI scans is meticulously collected from both the INMOL and General Hospital in Lahore, Pakistan, under the careful supervision of experienced radiologists. These medical experts ensured the quality and accuracy of the data, guaranteeing that each image met the necessary standards for research and analysis. With their guidance, the dataset provides a valuable resource for studying brain anatomy, pathology, and various medical conditions. The collaboration between these institutions and radiologists underscores the importance of rigorous data collection procedures in medical imaging research, facilitating advancements in diagnostic techniques and treatment strategies. The input image size is set to 416 × 416 pixels.

### 3.3. Preprocessing

In this step, preprocessing operations are performed on the collected data of the proposed technique. The preprocessing operations are required before analyzing the desired information of the original image geometrically. These enhancements include the correction of data to detect deviation and unwanted atmospheric noises, the elimination of images of nonbrain elements, and the conversion of data so that they are correctly reflected in the original image. The main problem in analyzing an image is noise, diffuse contrast, field bias (appearance of finely variable intensities in the network), and effects of partial evolution (voxels contribute to various types of networks) [40]. The proposed technique uses a balanced dataset with an equal number of images per class. Each class contained 500 images. The dataset contains images gathered from INMOL hospital along with open‐source images from Kaggle. The proposed technique uses a Gaussian filter with a kernel size of 3 × 3 and a standard deviation of 1.5 for image denoising. There are some image enhancement and filtering techniques which are applied in the preprocessing as follows: median filter, mean filter, and Gaussian filter. For YOLOv3, labeled bounding boxes are drawn on images for the best results.

### 3.4. Feature Extraction

In this step, the features are extracted from the preprocessed data. The extract of features includes reduced resources. One of the main problems in the search for complex data is the number of shared variables. The analysis using a large amount of memory and overlay can therefore be based on a similar algorithm. In the proposed technique, the statistical features and texture features based on the GLCM are extracted from the normal and tumor‐based MRI of the human brain. The details of extracted features by the proposed technique are given in the following subsequent sections.

#### 3.4.1. Extraction of Statistical Features

Statistical features include the features based on statistics which are as follows.

• *Mean*: The average of an image is calculated by dividing all pixel values of an image by the total number of pixels in an image. It is mathematically defined as follows:
(1) M=1m×n∑x=0m−1 ∑n=0n−1fx,y.
• *Median*: The median is the middle value among all pixel values in the image.
• *Kurtoses*: The shape of the probability distribution of any variable is indicated by the kurtosis parameter. For any variable, kurtosis is represented as *K*_urt_. It is mathematically defined as follows:
(2) Kurt=1m×n∑fx,y−M4SD4.
• *Covariance*: The covariance (denoted by *C*_ness_) is a measure of how two different random variables are simultaneous. It is the same as the variance, but when the variance tells you how a variable changes, the covariance tells you how two variables change together. It is mathematically defined as follows:
(3) Cness=12m+n∑x=0m−1 ∑y=0n−1fx,y.
• *Standard deviation*: It is the second central moment to the probability of an observed population and can serve as an estimation of the inhomogeneity. A higher value shows a better image intensity and contrast edges. The standard deviation (denoted by SD(*σ*)) is mathematically defined as follows:
(4) SDσ=1m×n∑x=0m−1 ∑y=0n−1fx,y−M2.
• *Variance*: The variance (denoted by *V*) is the average of squared differences from the mean. Variance measures how far the pixel values of an image are spread out. The variance is mathematically defined as follows:
(5) V=1m×n∑x=0m−1 ∑y=0n−1fx,y−M2.
• *Root mean square (RMS)*: The RMS is the square root of the arithmetic mean of the squares of the pixel values of the image. The RMC is mathematically defined as follows:
(6) RMS=1m×n∑x=0m−1 ∑y=0n−1fx,y.

#### 3.4.2. Extraction of GLCM Features

The proposed technique also uses texture features based on the GLCM. The GLCM is a statistical method that captures spatial relationships of pixel intensity values in an image. This matrix essentially describes how often different combinations of pixel intensity values occur in the image. The GLCM features utilized by the proposed technique are computed by analyzing the frequency of occurrence of pairs of pixel values at a specified spatial relationship (distance and angle) within the MRI image [41]. The GLCM features extracted by the proposed technique are as follows:

• **Correlation:** The correlation (denoted by *C*_orr_) describes the spatial dependencies of the pixels of the MRI image. It is mathematically defined as follows:
(7) Corr=∑x=0m−1 ∑y=0n−1x,yfx,y−MxMyσxσy.
• **Contrast:** The intensity part of a pixel and its effect on the MRI image is defined as a contrast. The contrast (denoted by *C*_on_) is mathematically defined as follows:
(8) Con=∑x=0m−1 ∑y=0n−1x−y2 fx,y.
• **Energy:** Energy (denoted by *E*_*n*_) can be defined as the number of repeatable pixels that can be measured. The energy is the limit to allow the similarity of the MRI images. It is mathematically defined as follows:
(9) En=∑x=0m−1 ∑y=0n−1f2x,y.
• **Homogeneity:** The homogeneity measures the closeness of the distribution of the pixels. The homogeneity (denoted by *H*) is mathematically defined as follows:
(10) H=∑x=0m−1 ∑y−0n−111+x−y2 fx,y.

#### 3.4.3. CSV File

In the proposed technique, every MRI image sample contains 12 values. Data samples are in the rows, and features are in the columns. After extracting all these features from the MRI images, a CSV file is compiled from these values which are used for the training of the ensemble classification by the proposed technique.

### 3.5. Classification

This section presents details of the training parameters and classification procedure used by the proposed technique. The proposed technique uses ensemble classification based on the SVM and KNN which leverages both global and local classification strengths to enhance the diagnostic accuracy of the brain tumor detection. The proposed technique built an ensemble classification model by training both SVM and KNN individually on the extracted features in the previous step. The SVM constructs a hyperplane for robust decision boundaries, while KNN classifies tumors based on proximity within the feature space. The proposed technique combines SVM and KNN through a two‐stage hybrid model. In this setup, the SVM classifier is first trained on the entire dataset to establish a global decision boundary. Subsequently, the KNN classifier is applied to data points that lie near the SVM’s decision boundary areas where the SVM’s confidence is lower. This strategy of the proposed ensemble classification allows KNN to refine classifications in regions where SVM may be uncertain, effectively reducing misclassifications near class boundaries. In the training phase of the ensemble classification model, parameters for the training of brain MRI images are carefully selected as they play a pivotal role in achieving optimal classification accuracy. The dataset is split into 70%–30% ratio for training and testing, respectively. By exploring various kernels, the radial basis function (RBF) is selected for SVM due to its optimal performance, alongside adjusting parameters like regularization (C), and kernel coefficient (gamma), the ensemble classification model can effectively delineate intricate patterns within the MRI data. Additionally, fine‐tuning parameters like class weights and tolerance ensure robustness against imbalanced datasets and convergence stability during optimization. These classifier predictions are then fused using a majority voting scheme, ensuring improved reliability and robustness by the proposed ensemble technique. In the majority voting scheme of the proposed ensemble classification technique, the final prediction of the ensemble classification is based on a simple majority of predictions made by individual classifiers (i.e., SVM and KNN). For a given test sample, each base classifier independently assigns a class label. The proposed ensemble classification then selects the class label that receives the most votes. This method is particularly effective in the proposed technique as the base classifiers are diverse and perform comparably well. The final ensemble prediction of the proposed ensemble technique is given as follows:

(11) y∧=modeySVM,yKNN,

where mode denotes the class with the highest frequency among the predictions (*y*_SVM_, *y*_KNN_). SVM and KNN both provide their individual predictions, and the majority vote class is selected as the final class by the proposed ensemble technique.

The performance of the proposed ensemble technique is validated using standard performance metrics like accuracy, sensitivity, specificity, and confusion matrices to confirm its effectiveness. By combining SVM’s capability to handle complex margins and KNN’s ability to classify based on similarity, the proposed technique based on the ensemble approach significantly enhances brain tumor detection, aiding medical professionals in making more precise diagnoses and treatment decisions. Through systematic experimentation and cross‐validation techniques, the ensemble classification model can be tailored to discern subtle nuances in brain MRI images, ultimately enhancing the diagnostic accuracy and clinical utility of the proposed technique.

For the training model with YOLOv3, training parameters are set precisely, and the same dataset is used for data augmentation like flipping and rotation. The input image size is set to 416 × 416 pixels. The model is trained using the Darknet framework, which is an open‐source neural network framework. The optimal training parameters like learning rate, batch size, and number of iterations are configured for the proposed technique. The learning rate was set to 0.001 and the batch size to 4, in accordance with the limited number of available training samples. The number of classes in the dataset is set to 1 by the proposed technique. The model is trained using backpropagation with stochastic gradient descent (SGD) optimization. It is reorganized iteratively by calculating the gradients of the loss function regarding the model parameters and altering the parameters to decrease the loss.

Classification mainly involves the actual detection of the tumor using different or one of the algorithms from supervised learning [42]. In the proposed technique, the aforementioned supervised ML algorithms have been used because the data is known as an output which we will try to predict. In the proposed technique, the aforementioned two classifiers (SVM and KNN) are used for ensemble classification, and their combined accuracy is 80.50% with ensemble classification and 97.80% with the YOLOv3 technique.

### 3.6. Postprocessing

**Figure 6:**
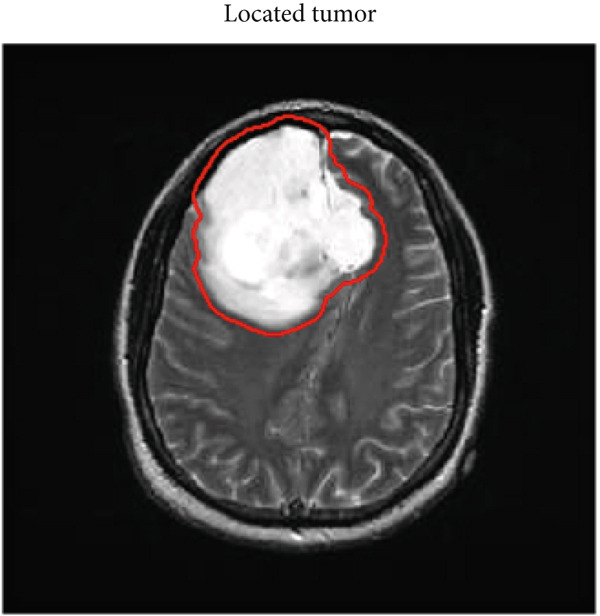
Result of tumor detection using the proposed technique based on the ensemble classification.

**Figure 7:**
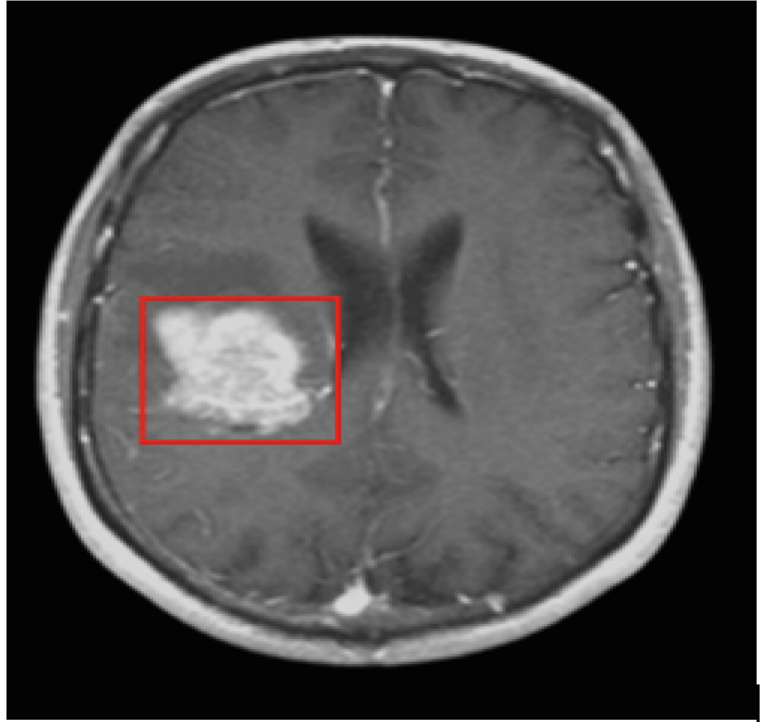
Result of tumor detection using the proposed technique based on the YOLOv3 technique.

This section presents details of the postprocessing of the proposed technique which includes the estimation of the area and location of the tumor within the MRI image. If the location and area are classified in the dataset then this post‐processing step is optional but if the location and area are not classified in the dataset, then we have to estimate the area and location of the tumor using an image processing technique. In the postprocessing phase, the labeled image is converted into a binary color scheme. After that, the boundary of the tumor region is extracted, and using the dilation, fill that tumor. After the filling, white pixels are counted, and using the pixel per millimeter formula, the number of pixels is converted into millimeter area, and then again, the boundary of a tumor is marked for estimation of the tumor location by the proposed technique based on the ensemble classification and YOLOv3 technique as shown in Figures 6 and 7, respectively.

### 3.7. Parallel Computing

Parallel computing refers to dividing a set of instructions for parallel execution. In this study, parallel processing is used to make the process fast; for this purpose, a GPU is used. To make the processing fast, the MRI image is given to the GPU, and the GPU extracts features and gives those features to the classifier for the classification of the MRI image. GPU coder in MATLAB is used to convert the MATLAB code into CUDA code and also provide the executable .M file that has the CUDA code which is executable in MATLAB. This file takes an input of an MRI image and extracts features using a GPU according to the aforementioned steps of the proposed technique.

## 4. Performance Metrics, Results, and Discussions

This section presents details of the performance evaluation metrics, results, and discussions of the proposed technique.

### 4.1. Performance Metrics

The proposed technique uses the following performance evaluation metrics to measure its performance. The detail of each metric is given in the following subsequent sections.

#### 4.1.1. Confusion Matrix

A confusion matrix is a matrix used to evaluate the performance of a classification model (or “classifier”) in a set of test data for which the actual values are known. It compares the predicted labels of the model with the actual labels of the data.

#### 4.1.2. Receiver Operating Characteristic (ROC) Curve

The ROC curve measures the performance of the classification problem in different threshold configurations. The ROC curve is plotted with true positive rate (TPR) against the false positive rate (FPR), where TPR is on the *y*‐axis and FPR is on the *x*‐axis.

#### 4.1.3. Mean Intersection Over Union (IoU)

The IoU is the relationship between the overlap region and the union area of the ground truth and predicted boundary box [43]. It can be defined mathematically as follows:

(12) IoU=intersection areaunion area.

**Figure 8:**
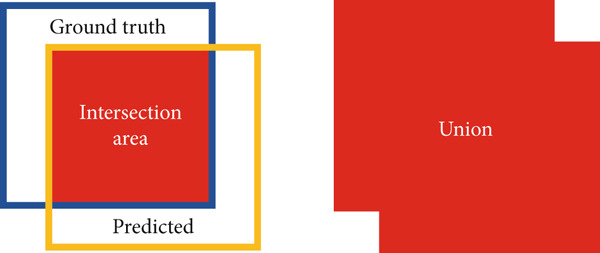
Intersection and union.

As shown in Figure 8, the union is the area of both the ground truth and predicted boundary boxes while the intersection area is the area of the overlap. For a good object detector, the intersection area should be higher. The maximum value for IoU can be 1, which is an ideal condition.

#### 4.1.4. Precision (P)

The P is also known as positive predictive value (PPV), and it measures the proportion of true positive predictions out of all positive predictions. It is calculated as follows:

(13) P=TPTP+FP,

where TP is the number of true positives and FP is the number of false positives.

#### 4.1.5. Recall (R)

The R is also known as sensitivity, and it measures the proportion of true positive predictions out of all actual positives. It is calculated as follows:

(14) R=TP TP+FN,

where TP is the number of true positives and FN is the number of false negatives.

#### 4.1.6. Accuracy

The accuracy is defined as the proportion of correctly classified instances out of the total number of instances [44–46].

### 4.2. Results and Discussions

This section presents the details of the experimental parameters and results of the proposed technique on different MRI for healthy and nonhealthy cases. The process diagram or flow diagram of the proposed technique is shown in Figure 5, and its results are presented in Table 3. The proposed technique, based on the YOLOv3, provides better computational efficiency and real‐time performance than the ensemble classification technique and cutting‐edge methods like Faster R‐CNN and U‐Net. In contrast to the ensemble technique proposed in this study, which depends on complicated feature fusion and several classifiers, YOLOv3 offers end‐to‐end detection in a single forward pass, lowering resource overhead and latency. Faster R‐CNN is more accurate, but its two‐stage detection pipeline is slower, while U‐Net’s pixel‐wise segmentation requires fine‐grained annotations, and it is computationally expensive, despite being precise. With its competitive accuracy and quick, automated tumor localization, the proposed YOLOv3 technique achieves the appropriate balance and is perfect for scaled clinical applications. For real‐world medical imaging applications, its lightweight design outperforms bulkier counterparts in terms of speed and usefulness, allowing integration into edge devices.

**Table 3:** Results of the proposed techniques in terms of the performance evaluation metrics.

**Performance metrics**	**Proposed technique based on the ensemble classification**	**Proposed technique based on the YOLOv3**
True positive (TP)	405	487
True negative (TN)	400	491
False positive (FP)	100	13
False negative (FN)	95	09
Precision	80.19%	97.40%
Recall	81.00%	98.18%
Accuracy	80.50%	97.80%

Several significant biological markers can be utilized to identify human brain tumors, including a study of the symptoms, biomarkers, imaging characteristics, cerebrospinal fluid (CSF) review, histology, and genetic testing. Some signs of a brain tumor may include headaches, seizures, or changes in cognitive function. The presence and type of a brain tumor can be determined by biomarkers, which are unique chemicals that can be found in blood, urine, or other bodily fluids. Imaging characteristics detected on MRI or CT scans, such as aberrant contrast enhancement, uneven boundaries, and edema (swelling) surrounding the tumor, can point to the presence of a brain tumor. The transparent fluid that surrounds the brain and spinal cord, known as CSF, can be examined to determine the presence of tumor cells and the concentrations of specific biomarkers. Histology is the process of examining tissue samples taken from biopsies under a microscope to determine the type and grade of the brain tumor. A genetic test can identify gene alterations or mutations that are linked to types of brain tumors. The detail of the experimental results of the proposed technique for healthy and nonhealthy cases is presented in the following subsequent sections.

#### 4.2.1. Healthy Case

**Figure 9:**
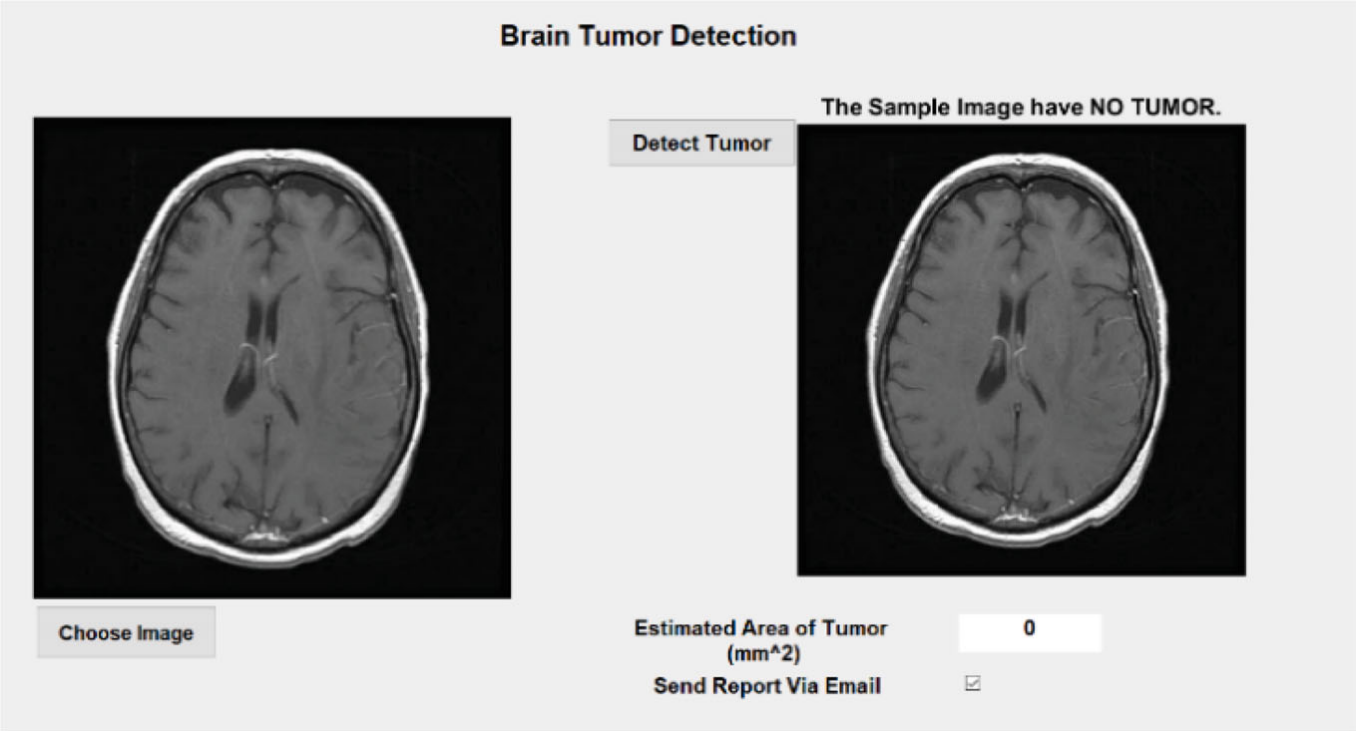
Result of no tumor detected using the proposed technique based on the ensemble classification (Sample 1).

If the test image is passed to a trained model that has no tumor, then the proposed technique marks it as healthy, and its results are shown in Figure 9 on two different samples of the human brain image.

**Figure 10:**
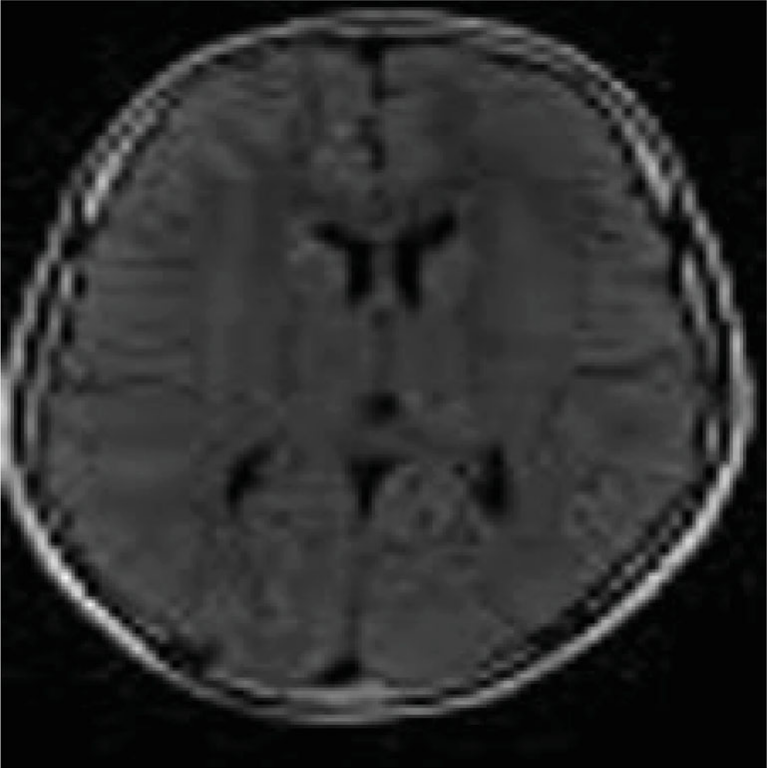
Result of no tumor detected using the proposed technique based on the YOLOv3.

Figure 10 shows the result of the proposed technique based on the YOLOv3 while detecting tumors from a normal brain MRI.

#### 4.2.2. Tumor Case

**Figure 11:**
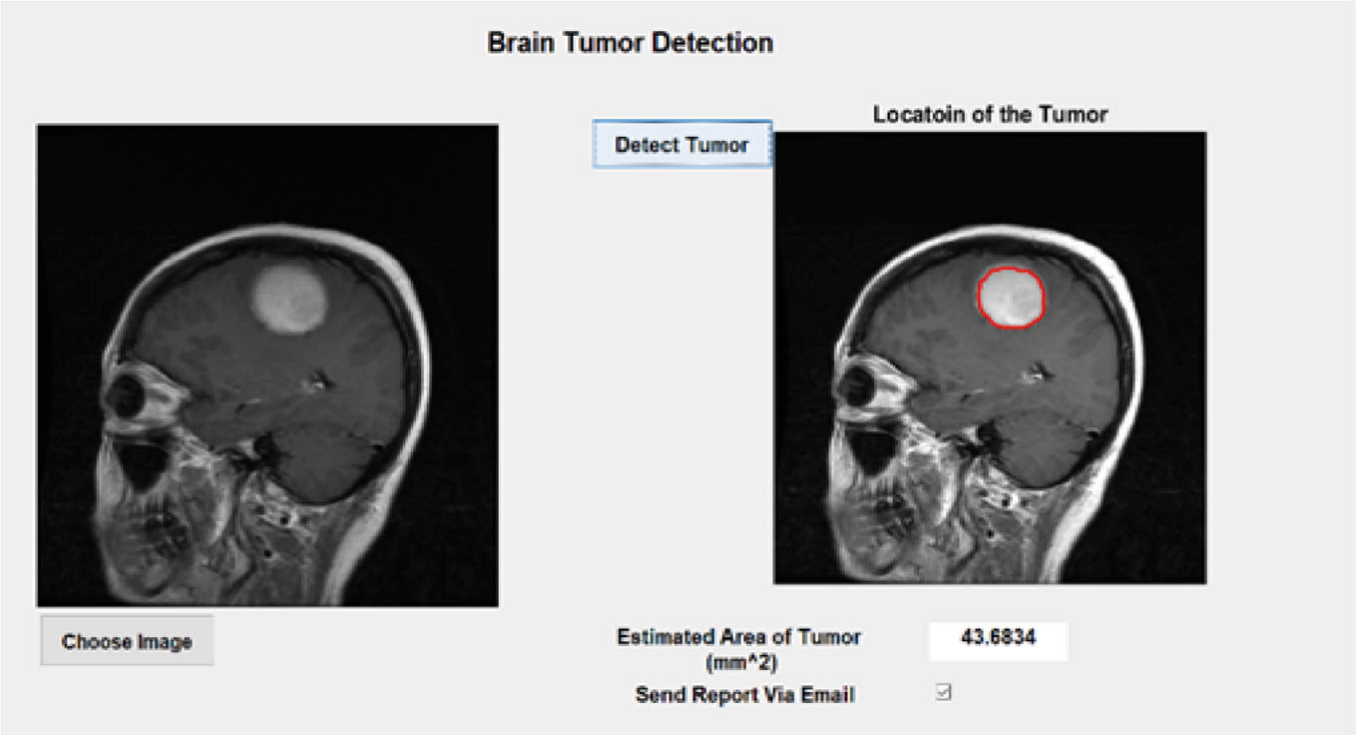
Result of detected tumor and its area using the proposed technique based on the ensemble classification (Sample 1).

**Figure 12:**
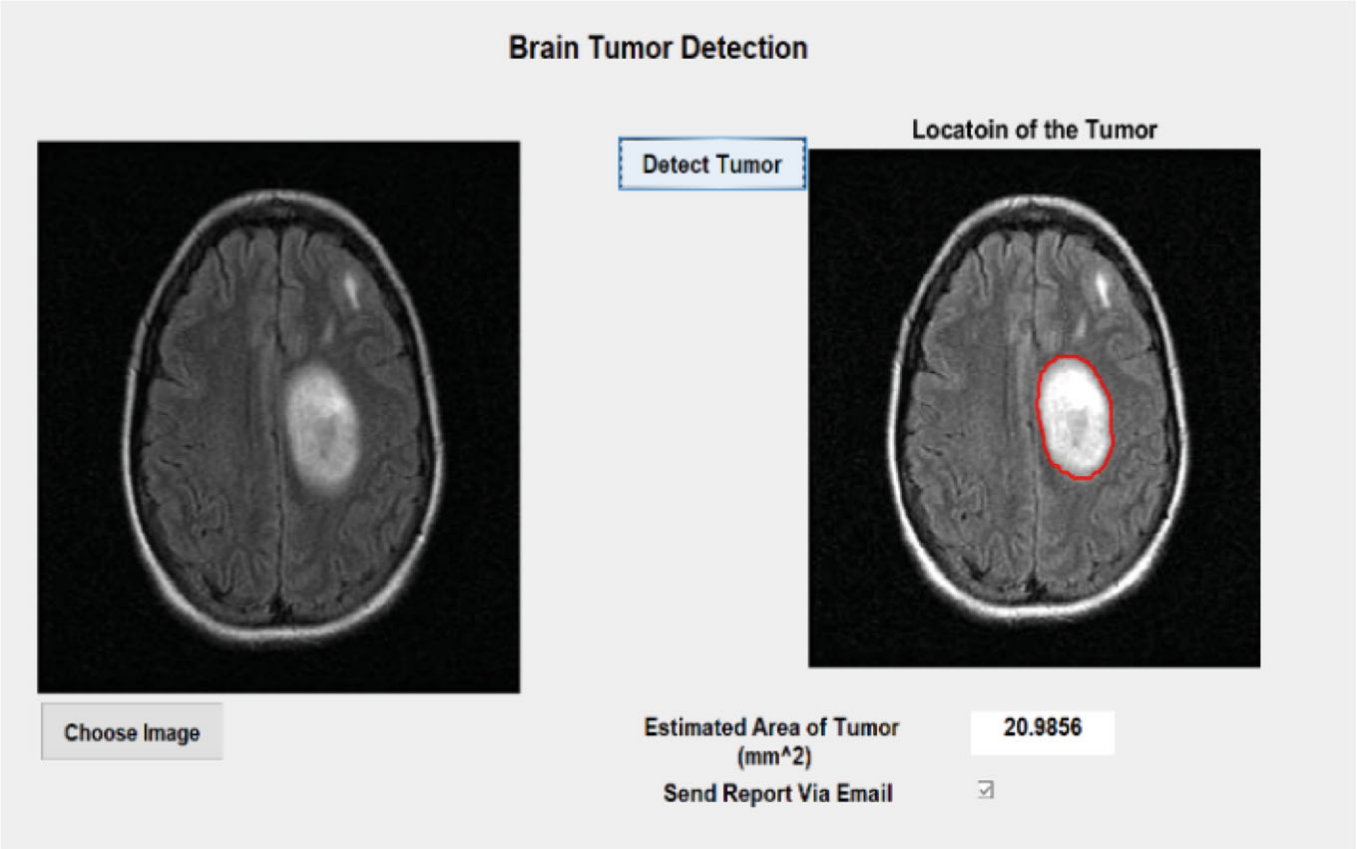
Result of detected tumor and its area using the proposed technique based on the ensemble classification (Sample 2).

When an MRI test image is passed to the proposed technique that has a tumor, then it marks as a tumor and estimates the area of the tumor. The results of the proposed technique are shown on two different sample MRI images of the human brain in Figures 11 and 12. The proposed technique based on the ensemble classification can detect brain tumors of sizes from 5 to 80 mm.

Figure 13a,b depicts the remarkable capability of the proposed technique based on the YOLOv3 in detecting brain tumors within MRI images. By utilizing the proposed technique based on the YOLOv3, the input image undergoes sophisticated analysis, resulting in precise localization and delineation of the tumor region through bounding boxes. Each bounding box encapsulates the detected tumor, providing a clear visual representation of its location and extent within the image. This robust detection capability not only aids in early diagnosis but also facilitates accurate assessment and monitoring of tumor progression.

Figure 13(a) Input MRI image. (b) Result of detected tumor using the proposed technique based on the YOLOv3.

**Figure:**
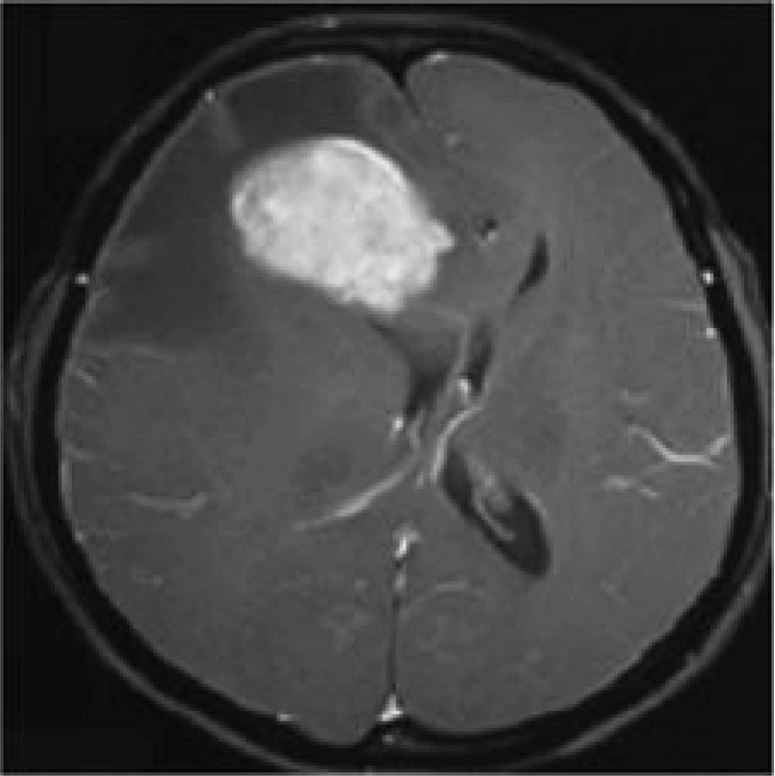
(a)

**Figure:**
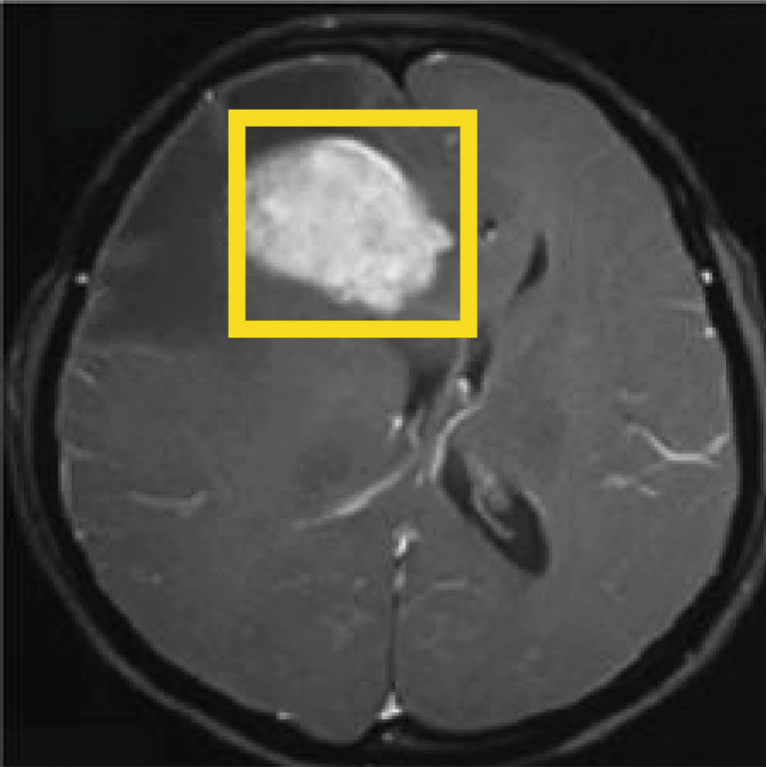
(b)

#### 4.2.3. Confusion Matrix

**Figure 14:**
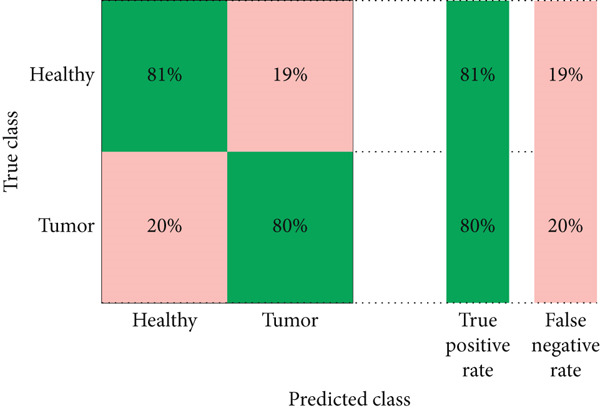
Confusion matrix of the proposed technique based on the ensemble classification.

**Figure 15:**
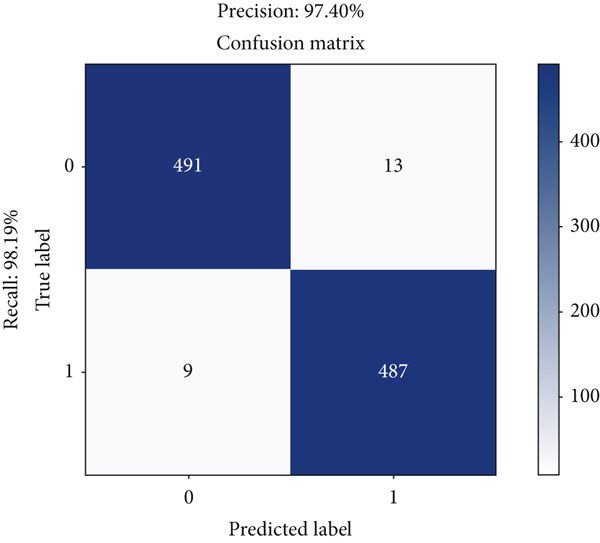
Confusion matrix of the proposed technique based on the YOLOv3.

The performance analysis of the proposed technique based on the ensemble classification and proposed technique based on the YOLOv3 is also performed in terms of a confusion matrix whose details are shown in Figures 14 and 15, respectively.

Table 3 presents the comparison between the two models of the proposed technique. The proposed technique based on the YOLOv3 outperforms the proposed technique based on the ensemble classification for effective detection of brain tumors in MRI images with 487 true positives compared to ensemble classification with 405 true positives. Similarly, the proposed technique based on the YOLOv3 also demonstrates superior P with only 13 false positives, while the proposed technique based on the ensemble classification records 100 false positives. This research’s focus solely on brain images restricts its applicability to other medical imaging modalities or types of tumors present in different anatomical regions. Additionally, the model’s inability to detect clots or other anomalies beyond tumors may limit its utility in comprehensive diagnosis and treatment planning. Furthermore, although YOLOv3 exhibits superior performance in object detection tasks compared to traditional ML algorithms like SVM, it may still struggle with detecting smaller or subtle abnormalities, particularly in cases with complex or ambiguous imaging features. Additionally, the reliance on locally available images with artifact issues may introduce bias or limitations in the dataset, potentially affecting the model’s generalizability to diverse patient populations or imaging settings.

#### 4.2.4. ROC Curves

**Figure 16:**
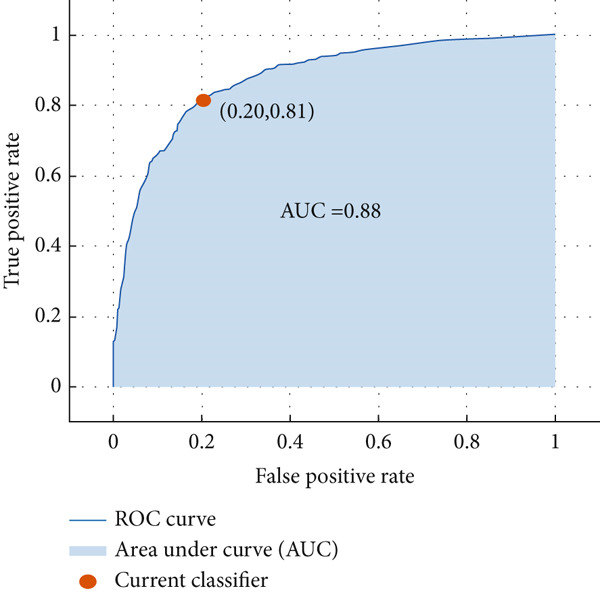
ROC curve of the proposed technique based on the ensemble classification.

**Figure 17:**
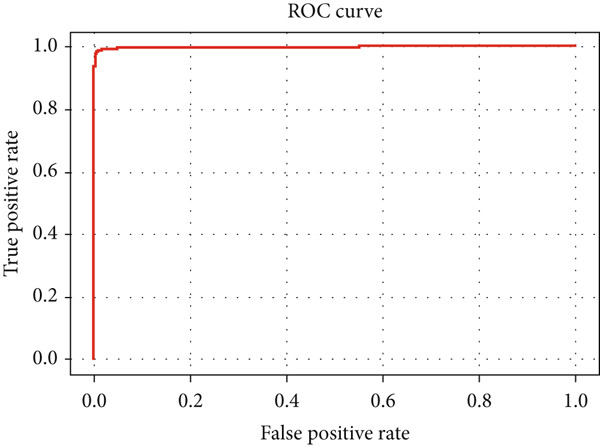
ROC curve of the proposed technique based on the YOLOv3.

The performance analysis of the proposed technique based on the ensemble classification and the proposed technique based on the YOLOv3 are also performed in terms of the ROC curves, and their details are shown in Figures 16 and 17, respectively. Comparing the ROC curves for the proposed technique based on the ensemble classification and the proposed technique based on the YOLOv3 reveals distinct performance characteristics between the two models in the context of tumor detection tasks. The ROC curve for ensemble classification showcases its efficiency in binary classification, with the curve illustrating the trade‐off between TPR (sensitivity) and FPR (specificity). The ROC curve for YOLOv3 demonstrates its proficiency in tumor detection. The YOLOv3 ROC curve displays a more intricate shape, reflecting its capability to adjust detection thresholds to optimize both true positive detections and false positive suppressions. Additionally, with higher AUC in the ROC curve of the proposed technique based on the YOLOv3 indicates superior discrimination ability in object detection scenarios.

#### 4.2.5. Mean IoU

Another important parameter of mean IoU is calculated to find how exactly the proposed technique based on the YOLOv3 detects the tumor location. The values of IoU are found for the 500 tumor‐containing images separately, and then, the mean IoU is found using the following equation:

(15) mIoU=∑1nIoUnn,

where *n* is the number of tumor images (500).

The mean IoU for the proposed technique based on the YOLOv3 is found to be 0.65. With a mean IoU of 0.65, the YOLOv3 model achieves a substantial level of agreement between its predictions and the actual object boundaries for tumor detection in MRI images. The YOLOv3 demonstrates its ability to accurately delineate object boundaries by measuring the overlap between predicted and ground truth bounding boxes. This metric provides a comprehensive assessment of the model’s spatial alignment and localization accuracy. The high mean IoU score of 0.65 solidifies YOLOv3’s efficacy in precisely identifying object regions within images, further enhancing its reliability for applications requiring precise object localization and segmentation. In contrast, ensemble classification, while effective, may struggle to achieve such fine‐grained delineations, highlighting the distinct advantages offered by DL approaches like YOLOv3 in object detection tasks.

#### 4.2.6. Computation Time

The proposed technique based on the YOLOv3 has a faster detection time compared to the proposed technique based on the ensemble classification. For a grayscale image of size 416 × 416 pixels, the computation time for the YOLOv3 is around 11 ms which is calculated on a computer having specifications of RTX 2060 Super with Core i7 9th generation CPU, 16 GB memory, and Microsoft Windows 8.1 (64 bit) operating system. However, ensemble classification is not inherently designed for object detection tasks like YOLOv3. It is used for binary classification tasks, with additional preprocessing and postprocessing steps, which impact overall inference time, whereas the computation time of the proposed technique based on the ensemble classification is around 230 ms with the same aforementioned hardware specifications. As it is not a real‐time detection task, accuracy is the key factor for the selection of the algorithm.

## 5. Conclusions and Future Work

In this research, novel models are proposed for brain tumor detection which uses ensemble classification and YOLOv3 to classify the healthy and tumor MRI brain images from the locally available images with artifact issues. After the successful classification, if there is a tumor in the MRI brain image, then the area and location are also estimated by highlighting a boundary against a tumor. To make this process fast, the proposed technique uses a built‐in CUDA platform. Furthermore, a promising innovation is a model that simultaneously recognizes and distinguishes normal scans and tumors in brain MRI. The comparison between the performance metrics of the ensemble classification model and the proposed technique based on the YOLOv3 model demonstrates a significant contrast in their capabilities for object detection tasks. While both models achieve commendable results, YOLOv3 consistently outperforms the ensemble classification model across all metrics. The proposed technique based on the YOLOv3 model exhibits superior P, R, and accuracy, with P reaching an impressive 97.40%, indicating its ability to accurately identify true positives while minimizing false positives. Its R rate of 98.18% signifies its proficiency in capturing a high proportion of actual positives. Additionally, the YOLOv3 model achieves an exceptional accuracy of 97.80%, indicating its overall effectiveness in correctly classifying both positive and negative instances. In contrast, the ensemble classification model, although respectable in its performance, falls notably short in P, R, and accuracy when compared to YOLOv3. These results highlight the advancement and efficacy of DL models like YOLOv3 in complex object detection tasks, showcasing their potential for real‐world applications where high P and R are paramount. In addition to the P, R, and accuracy metrics, the mean IoU further underscores the superior performance of YOLOv3 in object detection tasks. For future research work, combining multiple imaging modalities such as MRI, CT, PET, and functional MRI (fMRI) can provide a more comprehensive view of brain tumors, aiding in more accurate detection and classification in the proposed technique.

## Ethics Statement

This research study was approved by the UGS ethics approval committee (Approval No. 4132) of the Department of Electrical Engineering, University of the Punjab, Lahore.

## Consent

Written informed consent was obtained from all participants.

## Conflicts of Interest

The authors declare no conflicts of interest.

## Author Contributions

D. A.: data collection, conceptualization, methodology, formal analysis, and writing—original draft. Z.M.: data collection, preprocessing, validation, writing—review and editing, supervision, and project administration. A.U.: software development, visualization, and review and editing. A.F.: literature review, validation, and writing—review and editing. S.W.: review and editing, supervision, and project administration.

## Funding

No funding was received for this manuscript.

## Data Availability

The data that support the findings of this study are available from the corresponding author upon reasonable request.
